# Fertility of a spontaneous hexaploid male Siberian sturgeon, *Acipenser baerii*

**DOI:** 10.1186/1471-2156-15-5

**Published:** 2014-01-10

**Authors:** Miloš Havelka, Martin Hulák, Petr Ráb, Marie Rábová, Dietmar Lieckfeldt, Arne Ludwig, Marek Rodina, David Gela, Martin Pšenička, Dmytro Bytyutskyy, Martin Flajšhans

**Affiliations:** 1 University of South Bohemia in Ceske Budejovice, Faculty of Fisheries and Protection of Waters, South Bohemian Research Center of Aquaculture and Biodiversity of Hydrocenoses, Zátiší 728/II, 389 25 Vodňany, Czech Republic; 2 Laboratory of Fish Genetics, Institute of Animal Physiology and Genetics, Czech Academy of Sciences, 277 21 Liběchov, Czech Republic; 3 Department for Evolutionary Genetics, Leibniz Institute for Zoo and Wildlife Research, 10324 Berlin, Germany

**Keywords:** Acipenseridae, Polyploidy determination, Sperm quality, Autopolyploidization, Triploidization

## Abstract

**Background:**

Evolution of sturgeons and paddlefishes (order Acipenseriformes) is inherently connected with polyploidization events which resulted in differentiation of ploidy levels and chromosome numbers of present acipenseriform species. Moreover, allopolyploidization as well as autopolyploidization seems to be an ongoing process in these fishes and individuals with abnormal ploidy levels were occasionally observed within sturgeon populations. Here, we reported occurrence of Siberian sturgeon (*Acipenser baerii*) male with abnormal ploidy level for this species, accessed its ploidy level and chromosome number and investigate its potential sterility or fertility in comparison with normal individuals of sterlet (*A. ruthenus*), Russian sturgeon (*A. gueldenstaedtii*) and Siberian sturgeon (*A. baerii*).

**Results:**

*Acipenser ruthenus* possessed 120 chromosomes, exhibiting recent diploidy (2n), *A. gueldenstaedtii* and *A. baerii* had ~245 chromosomes representing recent tetraploidy (4n), and *A. baerii* male with abnormal ploidy level had ~ 368 chromosomes, indicating recent hexaploidy (6n). Genealogy assessed from the *mtDNA control region* did not reveal genome markers of other sturgeon species and this individual was supposed to originate from spontaneous 1.5 fold increment in number of chromosome sets with respect to the number most frequently found in nature for this species. Following hormone stimulation, the spontaneous hexaploid male produced normal sperm with ability for fertilization. Fertilization of *A. baerii* and *A. gueldenstaedtii* ova from normal 4n level females with sperm of the hexaploid male produced viable, non-malformed pentaploid (5n) progeny with a ploidy level intermediate to those of the parents.

**Conclusion:**

This study firstly described occurrence of hexaploid individual of *A. baerii* and confirmed its autopolyploid origin. In addition to that, the first detailed evidence about fertility of spontaneous hexaploid sturgeon was provided. If 1.5 fold increment in number of chromosome sets occurring in diploids, resulted triploids possess odd number of chromosome sets causing their sterility or subfertility due to interference of gametogenesis. In contrast, 1.5 fold increment in number of chromosome sets in naturally tetraploid *A. baerii* resulted in even number of chromosome sets and therefore in fertility of the hexaploid specimen under study.

## Background

Evolution of vertebrate genomes was possibly associated with three episodes of whole-genome duplication. The first occurred at the origin of vertebrates and another at the origin of gnathostomes, the 2R hypothesis [1,2]. The third, 3R, is suggested to have occurred in fin-rayed teleostean fishes after their divergence from the earliest lineage of actinopterygians, sturgeon and paddlefish of the extant order Acipenseriformes [3-6].

Sturgeon, paddlefish, the fishes of the genera *Psephurus*, *Polyodon* (Acipenseriformes: Polyodontidae), *Acipenser, Huso, Scaphirhynchus*, and *Pseudoscaphirhynchus* (Acipenseriformes: Acipenseridae) provide the most remarkable examples of evolution of ploidy levels among vertebrates [7]. Independent of the two ancient genome duplication rounds, several lineage-specific duplication events occurred in sturgeons and paddlefishes [8,9].

Within the most recent vertebrates, sturgeon is among the species with a large number of chromosomes. Nowadays, several well divided groups of acipenseriform species can be recognized depending on DNA content and the number of chromosomes in their cell nuclei. They include species with ~120, ~240 and ~360 chromosomes, corresponding to elevated DNA content [10]. Despite this widely respected division, ploidy status of Acipenseriformes often remains unresolved with conflicting opinions [10,11]. Recent investigations suggest two scales of ploidy levels in Acipenseriformes: the ‘evolutionary scale’, which presumes tetraploid - octaploid - dodecaploid relationships among species, and the ‘recent scale’, which presumes diploid–tetraploid–hexaploid relationships [11].

Probably due to the polyploid nature of their genomes, sturgeon of differing ploidy levels hybridize both in nature [e.g. [12-14] and in captivity [15,16], suggesting mutual fertility of various sturgeon species irrespective of ploidy level. Some authors [17-19] assumed hybrids of parent species having different ploidy levels to be sterile. The same generally applies to fertility of fishes with an odd number of chromosomes, such as autotriploids in which meiosis is seriously affected because three homologous chromosomes cannot pair during the zygotene stage of prophase I, interfering with gonad development and gametogenesis [20].

In the present study, we reported the occurrence of a spontaneous hexaploid male among hatchery stock of Siberian sturgeon, *Acipenser baerii*, a species of recent tetraploid level 4n with ~245 chromosomes; its experimental hybridization with normal females of *A. baerii* and *A. gueldenstaedtii*; and analysis of resulting viable progeny. The main aims of this study were to accessed chromosome number and ploidy level of analyzed individuals and investigate potential sterility or fertility of observed spontaneous hexaploid male of *A. baerii.*

## Results and discussion

### Confirmation of ploidy levels of analyzed individuals

**Figure 1:**
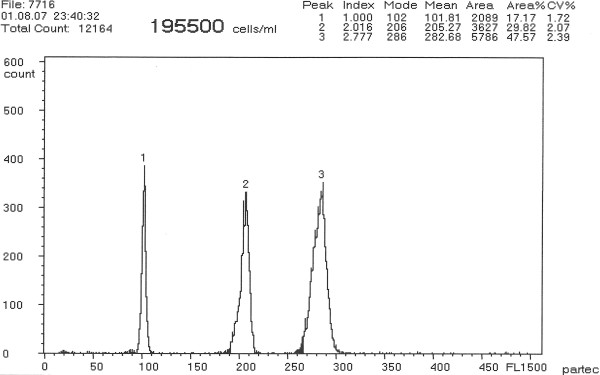
**Flow cytometric histogram showing relative DNA content in blood cells of sterlet (*A. ruthenus*; peak 1), Siberian sturgeon (*A. baerii*; peak 2) and spontaneous hexaploid Siberian sturgeon (*A. baerii*; peak 3).**

**Figure 2:**
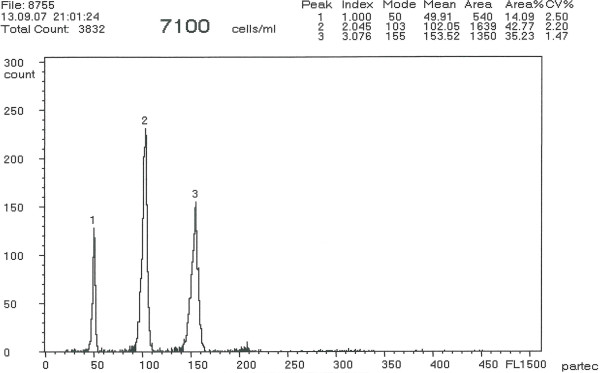
**Flow cytometric histogram showing relative DNA content in sperm of sterlet (*A. ruthenus*; peak 1), Siberian sturgeon (*A. baerii*; peak 2) and spontaneous hexaploid Siberian sturgeon (*A. baerii*; peak 3).**

**Figure 3:**
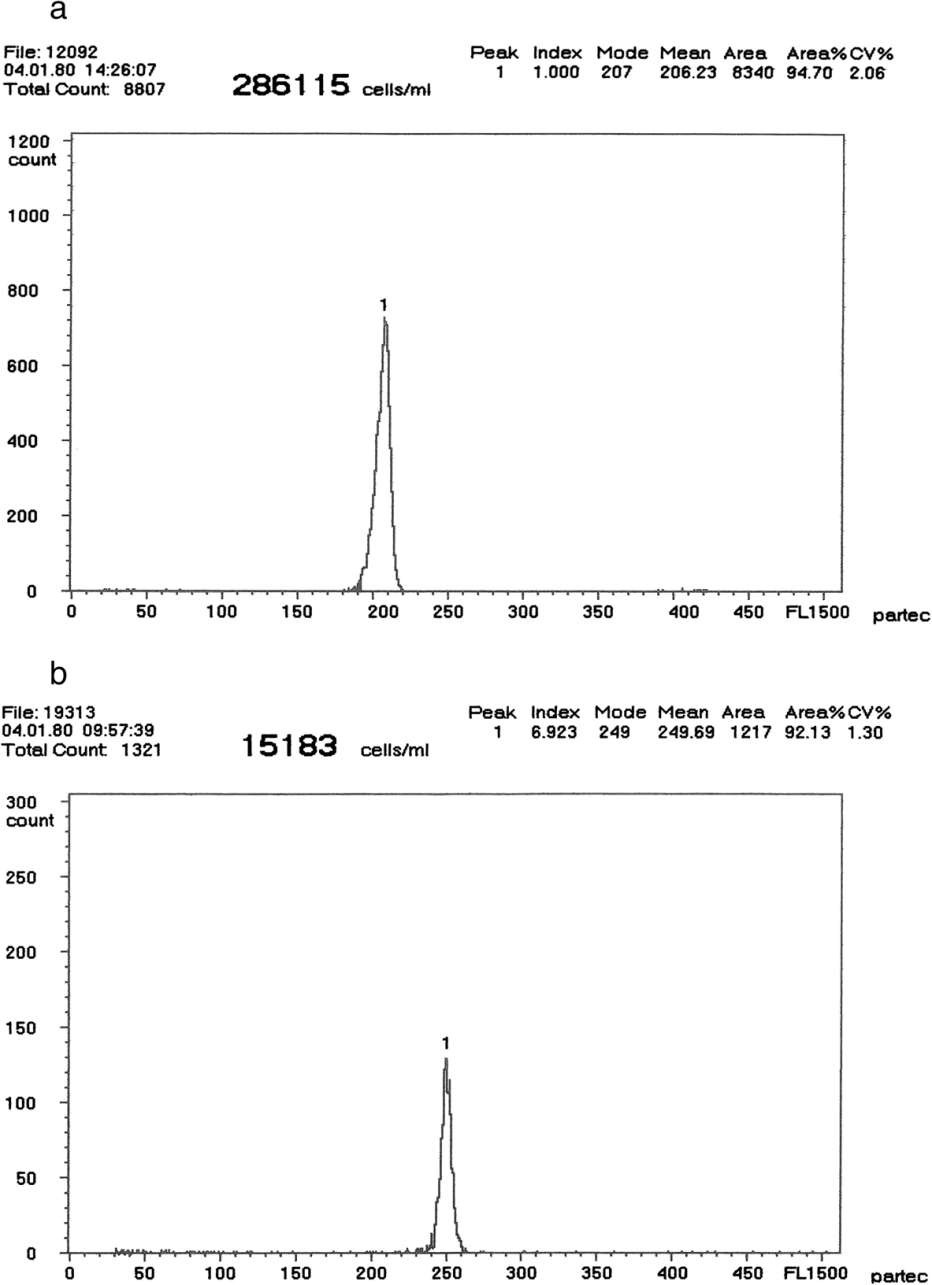
**Flow cytometric histogram showing relative DNA content in blood cells of juveniles from *A. baerii *** **and *A gueldenstaedtii *** **purebreeding (a) and hybridizations of the normal x spontaneous hexaploid *A. baerii *** **and the normal *A. gueldenstaedtii *** **x spontaneous hexaploid *A. baerii *** **(b).**

Flow cytometry revealed the erythrocyte relative DNA content of the 5 males of *A. ruthenus* standard to be diploid (2n; Figure 1, peak 1), 1 male and 1 female of *A. baerii* to be tetraploid (Figure 1, peak 2) and one specimen of *A. baerii* to be 1.38-fold that of the tetraploids (Figure 1, peak 3). Sperm of analyzed males had average relative DNA content equivalent to haploidy (1.0n; Figure 2, peak 1), diploidy (2.0n; Figure 2, peak 2), and triploidy (3.0n; Figure 2, peak 3), respectively. The erythrocyte relative DNA content of the *A. gueldenstaedtii* male and female under study was also tetraploid. The coefficient of variation (C_V_) in both erythrocyte and sperm relative DNA content was below or equal to 2.5% for all specimens. The relative DNA content in juveniles from purebreeding of *A. baerii* and *A gueldenstaedtii* (Figure 3a), from the normal x spontaneous hexaploid *A. baerii* and from the normal *A. gueldenstaedtii* x spontaneous hexaploid *A. baerii* hybridizations (Figure 3b) revealed 100% normal ploidy in the purebred juveniles compared to 100% intermediate ploidy level of the F_1_ hybrid juveniles. This was in agreement with widely accepted theory that hybrids resulted from hybridization between individuals with different chromosome numbers and ploidy levels were considered to have chromosome number and ploidy level intermediate to those of parental individuals [21].

Image cytometry analysis revealed mean erythrocyte nuclear area for *A. ruthenus* (19.39 ± 1.43^a^ μm^2^) confirming diploidy, *A. baerii* (29.95 ± 1.30^b^ μm^2^) and *A. gueldenstaedtii* (30.1 ± 1.05^b^ μm^2^) confirming tetraploidy and one specimen of *A. baerii* (39.59 ± 3.56^c^ μm^2^) to be 1.32-fold that of the tetraploids. These results correspond to already published data for sturgeons of higher ploidy levels [22]. These authors confirmed that the erythrocyte nuclear area did not increase linearily with increasing ploidy levels but that the DNA appeared to be more densely packed.

**Figure 4:**
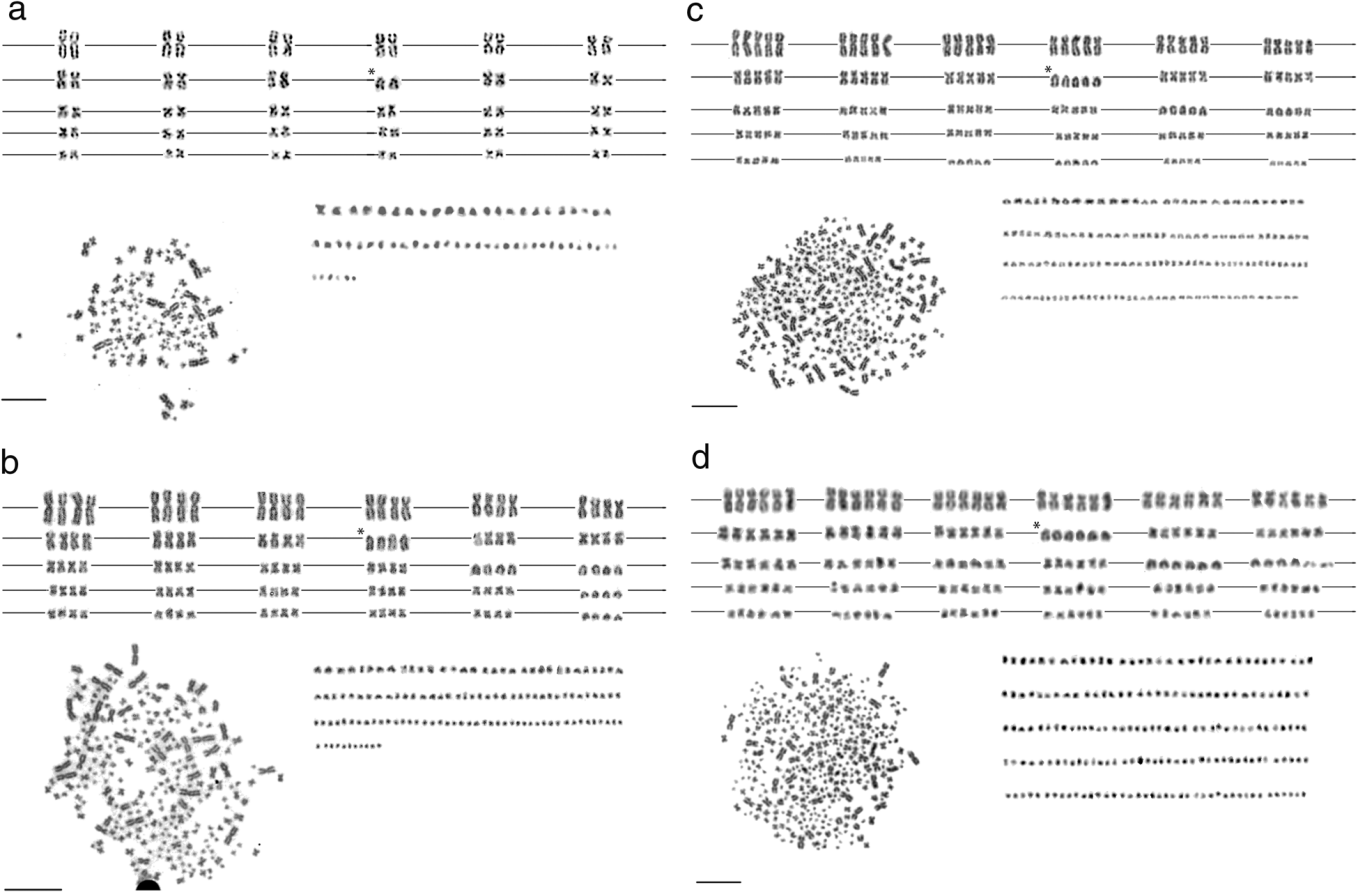
**Metaphase cell and corresponding karyotype arranged from Giemsa-stained chromosomes of (a) sterlet *Acipenser ruthenus *(2n = 120), (b) Siberian sturgeon *A. baerii *(2n ~ 245), (c) hybrid of A. baerii (2n ~ 245), x spontaneous hexaploid A. baerii (2n ~ 368) resulting in (2n ~ 300) and (d) spontaneous hexaploid A. baerii (2n ~ 368); asterisks denotes „acipenserine“ cytotaxonomic marker, i. e. the group of the largest acrocentric chromosomes.** Bars equal 10 μm.

Chromosome analysis of individuals under study revealed *A. ruthenus* specimens with chromosome number 2n = 120, *A. baerii* and *A. gueldenstaedtii* with modal chromosome number 2n ~ 245, while the *A. baerii* specimen with higher ploidy level exhibited chromosome number 2n ~ 368. Except for microchromosomes, all chromosomes of *A. ruthenus* could be paired (Figure 4a). Those of *A. baerii* grouped in quadruplets (Figure 4b), and those of the *A. baerii* specimen with higher ploidy level grouped into hexaplets (Figure 4d), indicating 1.5 fold increment in number of chromosome sets with respect to the number most frequently found in nature for a given species*.* If occurring in diploid species, this phenomenon is generally known as triploidization [19]. However, considering the tetraploid status of *A. baerii,* this phenomenon lead to the formation of hexaploidy.

Karyotype analysis of hybrid specimens of the normal x spontaneous hexaploid *A. baerii* revealed modal number 2n ~ 300 chromosomes, with macrochromosomes that grouped into apparent pentaplets (Figure 4c). The same ploidy level of progeny and karyotype structure resulted from crossing of the normal *A. gueldenstadtii* female x spontaneous hexaploid *A. baerii* male.

Ploidy levels of *A. baerii* and *A. gueldenstadtii* females used for cross-breeding corresponded to ~245 chromosomes as previously reported for these species [11]. Chromosome number 2n =120 of *A. ruthenus* used as ploidy standard also corresponded to findings of a number of other cytogenetic studies [23,24]. Ploidy level of progeny resulting from crossing *A. baerii* and *A. gueldenstaedtii* with the spontaneous hexaploid *A. baerii* male was intermediate between the ploidy levels of parents at ~300 chromosomes. This observation was in accordance with findings of Gorshkova et al. [21] and in contrast to the report of Arefjev et al. [25] who demonstrated non-intermediate karyotypes in progeny of hybridization of beluga, *Huso huso*, and *A. gueldenstaedtii*. The control mating of normal ploidy level *A. baerii* and *A. gueldenstaedtii* yielded progeny of the same ploidy level as the parents. Our results clearly demonstrated that sturgeons with different ploidy levels readily interbreed, as assumed for various combinations of sturgeon species [13].

### Confirmation of origin of the hexaploid male *A. baerii*

The nucleotide sequence of the *A. baerii* spontaneous hexaploid male was closely similar (99%, E = 0.0) to *A. baerii* haplotype GenBank: FJ843094.1 [26]. A maximum of three variable sites were detected in common with *A. baerii* sequences GenBank: EU185048, EU185049 and EU185051 [15]. Similar results were obtained for the *A. baerii* female. The *A. gueldenstaedtii* female had mtDNA haplotype closely similar (97%, E = 0.0) to *A. gueldenstaedtii* haplotype GenBank: FJ843096 [26].

Sturgeons with abnormal ploidy levels have been observed in wild populations [15,27] and in aquaculture [17], and interspecific hybridization has been considered the most probable origin of these. Individuals with abnormal ploidy levels can also arise through autopolyploidization mechanisms such as spontaneous diploidization of the maternal chromosome set (SDM) [28-30] or by polyspermic fertilization [10]. The SDM is a well-known event in fish, and natural autopolyploids resulting from fusion of a haploid sperm pronucleus with an SDM oocyte have been described [28-32].

Because there is no sturgeon species with a higher ploidy level and chromosome number than *A. brevirostrum* (6n ~372 chromosomes [33]), the hybrid origin of the *A. baerii* hexaploid might seem improbable. Bearing in mind that SDM of a tetraploid species leads to hexaploidy, the origin of the 1.5 fold increment in number of chromosome sets in genome of the observed specimen might be derived from autopolyploidization, specifically by the junction of pronuclei in a diploid egg, as a result of suppressing the second meiotic division, i.e. by the SDM mechanism. This statement is supported by findings of individuals with abnormal ploidy level among progeny obtained from artificial propagation of *A. mikadoi*[34]. Furthermore, these individuals showed a modal chromosome number of 360-402 in somatic cells [35], similarly to the hexaploid described in our study. The occurrence of individuals with abnormal ploidy level has also been detected in normal fertilized eggs of the bester (hybrid of *H. huso* and *A. ruthenus*) and SDM caused by egg over-ripening was suggested as the most probable cause [36]. Finally, spontaneous autopolyploids of *A. transmontanus* have also been recently reported [37]. These findings are contradictory to conclusions of Vasiľev [10] who stated that occurrence of spontaneous egg diploidization is close to zero in acipenserids.

### Confirmation of fertility of the hexaploid male *A. baerii* via sperm characteristics

Data on sperm volume per 1 kg M_B_, numbers of spermatozoa in 1 ml sperm, viability of spermatozoa and their motility and velocity are given in Table 1. The mean velocity of spermatozoa 15 s after activation varied between normal individuals and spontaneous hexaploid male *A. baerii* (169.66 vs. 152.04 μm s^-1^, respectively). Velocity of spermatozoa of *A. ruthenus* was 159 μm s^-1^, similar to the spontaneous hexaploid *A. baerii*. The evaluation of spermatozoa motility of hexaploid *A. baerii* showed normal state of sperm (100 % of motile spermatozoa). Sperm viability assay revealed similar rates of live spermatozoa (98.76 ± 0.36%, 95.93 ± 2.58%, and 93.47 for the *A. ruthenus*, normal *A. baerii*, and spontaneous hexaploid *A. baerii* males, respectively).

**Table 1:** **Relative volume of sperm kg**^**-1 **^**body mass (M**_**B**_**), concentration and viability of spermatozoa (spz) and motility and velocity 15 s post activation (mean ± SD) in sperm of *A. ruthenus*, *A. gueldenstaedtii, A. baerii *** **and of the spontaneous hexaploid *A. baerii***

**Males**	**No. of Fish**	**Relative volume of sperm (ml kg**^**-1 **^**M**_**B**_**)**	**Concentration (10**^**9 **^**ml**^**-1**^**)**	**Viability (% live spz)**	**Motility (% motile spz)**	**Velocity (μm s**^**-1**^**)**
*A. ruthenus*	6	13.57 ± 4.69	1.75 ± 0.46	98.76 ± 0.36	87.90 ± 12.10	159.00 ± 19.25
*A. gueldenstaedtii*	6	8.6 ± 4.6	1.54 ± 0.80	92.56 ± 3.2	90.60 ± 9.20	165.20 ± 15.72
*A. baerii*	6	22.14 ± 7.07	1.52 ± 1.88	95.93 ± 2.58	95.24 ± 9.85	169.66 ± 8.69
*A. baerii* spontaneous hexaploid	1	24.29	2.35	93.47	100.00	152.04

The observed *A. baerii* hexaploid produced similar volume of sperm as the diploid specimens. All sperm parameters observed in this experiment were within the range reported by other authors [38-41]. High rates of spermatozoa motility indicated high quality of sperm and corresponded with high viability. The initiation of spermatozoa velocity (in 15 sec) of the hexaploid *A. baerii* was lower than reported for males of normal ploidy level, as also observed by Pšenička et al. [42]. The authors considered the higher relative DNA content and larger size of heads of the hexaploid *A. baerii* spermatozoa to be the cause of the difference. Nevertheless this slower movement of spermatozoa did not affect successful fertilization.

### Confirmation of fertility of the hexaploid male *A. baerii* via analyses of its progeny

Experimental fertilization of ova of the normal *A. baerii* and *A. gueldenstaedtii* female with sperm of the spontaneous hexaploid *A. baerii* resulted in hatching rates of 64.16 ± 2.60% and 36.64 ± 2.06%, respectively, similar to that of *A. baerii* and *A. gueldenstaedtii* pure-breeding (50.56 ± 8.19% and 39.82 ± 3.64%). No apparent increase in the percent of malformed non-viable larvae was observed in either cross-breeding experiment.

Parental genotypes and observed genotypes of progeny at informative locus *Aox45* from experimental crossbreeding (Table 2) confirmed Mendelian segregation patterns in all specimens analyzed. The inheritance of parental alleles of the normal female *A. gueldenstaedtii* and spontaneous hexaploid male *A. baerii* were clearly evident at highly informative locus *Aox45*. The observed genotypes were predominately composed of two maternal and three paternal alleles while locus *Afu68* displayed apparently double diploid segregation of alleles. This was in agreement with Ludwig et al. [8], who supposed this locus duplicated in species with ~ 250 chromosomes. The genotypes observed at all analyzed loci are listed in electronic Additional file 1.

**Table 2:** **Genotyping results at locus *Aox45 *** **for hybridization of *A. gueldenstaedtii *** **female and *A. baerii *** **spontaneous hexaploid male**

**Sample**	**Allele 1**	**Allele 2**	**Allele 3**	**Allele 4**	**Allele 5**	**Allele 6**	**Allele 7**	**Allele 8**	**Allele 9**
*A. gueldenstaedtii* female	121				142	145			157
*A. baerii* spontaneous hexaploid male		124	127	136	142		148	151	
progeny 1	121	124				145	148	151	
progeny 2	121	124		136	142			151	
progeny 3				136	142		148		157
progeny 4			127		142		148		157
progeny 5		124			142			151	157
progeny 6	121	124	127			145		151	
progeny 7	121	124				145	148	151	
progeny 8		124				145	148	151	157
progeny 9			127		142		148		157
progeny 10		124	127		142	145			157
progeny 11	121	124	127	136					157
progeny 12	121	124			142			151	157
progeny 13		124	127		142			151	157
progeny 14	121	124		136	142			151	
progeny 15		124		136	142				157
progeny 16	121	124		136	142		148		
progeny 17	121		127		142	145		151	
progeny 18			127		142	145	148		157
progeny 19	121	124		136	142			151	
progeny 20	121	124	127			145		151	
progeny 21		124	127		142			151	157
progeny 22			127		142		148		157

Fertilization of ova of *A. baerii* and *A. gueldenstaedtii* with normal ploidy with sperm of the spontaneous hexaploid *A. baerii* produced fully viable progeny with ploidy level intermediate to those of the parents. This demonstrated full fertility of the spontaneous hexaploid male. Despite the fact that this specimen has originated by mechanism analogous to spontaneous autotriploidization, where the triploids are generally considered sterile or subfertile due to interference with gametogenesis, meiosis in the hexaploid male *A. baerii* under study was not affected by this factor. The 1.5 fold increment in number of chromosome sets in tetraploid *A. baerii* resulted in hexaploidy and homologous chromosomes could apparently group in triplets during the zygotene stage of prophase I during spermatogenesis, as was evident from segregation pattern of microsatellite alleles at representative locus *Aox 45* (Table 2).

## Conclusion

We confirmed the spontaneous hexaploid status of the examined *A. baerii* male, i.e. corresponding to ~368 chromosomes of the somatic cells and triploid sperm, a characteristic which has not been reported in other sturgeon species so far.

This study demonstrated that a hexaploid sturgeon male may be fertile owing to its polyploid ancestry and release viable and motile spermatozoa, and that these might have full fertilization potential resulting in a hatching rate similar to that of purebred sturgeon and produce viable progeny with ploidy levels intermediate between those of the parents. Based on these findings, it might be assumed that (auto)polyploids of other sturgeon species could be fertile as well. In light of this phenomenon, it is necessary to evaluate an impact of this event on sturgeon broodstocks in aquaculture, especially if these fishes are used for supportive breeding in reintroduction programs.

## Methods

This study was carried out in strict accordance with the Czech Law n. 246/1992 about “Animal welfare”. Authors possess a testimony according to §17 of Law 246/1992 about “Animal welfare”. Protocols have undergone the ethical review process by the University of South Bohemia animal care committee (PP3/FROV/2012; in Czech). Moreover, this was specifically approved by University of South Bohemia animal care committee. All surgery was performed under the clove oil anesthesia, and all efforts were made to minimize suffering.

### Fish and breeding details

Parental fish originated from the hatchery of the Faculty of Fisheries and Protection of Waters, University of South Bohemia. One 15-year-old *A. baerii* male [1.19 m total length (L_T_)_,_ 9.30 kg body mass (M_B_)], suspected of being spontaneous hexaploid after cytometric examination; one 14-year-old female *A. baerii* (1.15 m L_T_, 9.50 kg M_B_); and one 14-year-old female *A. gueldenstaedtii* (1.40 m L_T_, 13.60 kg M_B_) were used for the experiment. Fifteen-year-old male *A. baerii* and *A. gueldenstaedtii* (1.05 m L_T_ and 8.25 kg M_B_; 0.90m L_T_ and 7.00 kg M_B_, respectively) were used for controlled breeding with both females. Five sterlet males, *Acipenser ruthenus*, (0.51 ± 0.15 m L_T_; 0.65 ± 0.10 kg M_B_) were used as the diploid standard for flow cytometry and karyotyping. Fish were kept in 5 m^3^ indoor tanks supplied with re-circulating water at 14°C for 7 days prior to hormone stimulation. Before handling, fish were anesthetized by immersion in 0.07 ml l^-1^ clove oil [43]. Other 6 males of *A. baerii, A. gueldenstaedtii* and *A. ruthenus* were used for comparison of sperm quality parameters with those of hexaploid *A. baerii*.

Males were stimulated with an intramuscular injection of 4 mg kg^-1^ M_B_ carp pituitary suspension in physiological saline 36 h before expected sperm sampling [44].

Females were stimulated with an intramuscular injection of 0.5 mg kg^-1^ M_B_ carp pituitary suspension in physiological saline 42 h before expected ovulation, and again 12 h later with 4.5 mg kg^-1^ M_B_ of the same suspension [44]. Ovulated eggs for the cross-breeding experiments were collected after microsurgical incision of oviducts as described by Štěch *et al.*[45]. Fin clips were collected from all parent fish and stored in 96% ethanol.

### Sperm quantity and quality

Sperm was collected from the seminal duct, using a 5 mm diameter plastic cannula, into a 100 ml tissue culture flask following the protocol of Gela *et al.*[44]. Samples were maintained on crushed ice at 0 to 4°C. Sperm volume and spermatozoa concentration, motility, and velocity were assessed according to Linhart *et al.*[46]. Spermatozoa viability (% live) was determined by epifluorescence microscopy of dual-stained sperm DNA [47]. Wherever applicable, samples were processed in triplicate. The means and standard deviations for sperm characteristics of diploid *A. ruthenus*, tetraploid *A. gueldenstaedtii* and tetraploid *A. baerii* males were calculated from values of each individual.

### Fertilization experiment

Eggs of *A. baerii* and *A. gueldenstaedtii* were inseminated with sperm from the supposed spontaneous hexaploid *A. baerii*. Pure-breeding of *A. baerii* and *A. gueldenstaedtii* males and females was conducted as control. Eggs were put into plastic beakers in 50 g aliquots, which were placed on a shaking table with constant 200 rpm and 10 mm deflection. Each aliquot was inseminated with 1.5 ml of sperm and activated with 200 ml dechlorinated tap water at 15.0°C. After 2 min, fertilized eggs from each aliquot were separately distributed into 200 cm^3^ incubator cages, supplied with UV sterilized re-circulating tap water at 15.0°C, 9 mg l^-1^ O_2_ in triplicate. During incubation, eggs and hatched larvae were counted, and dead eggs were counted and removed. Hatching rate was computed as described by Linhart *et al.*[48]. Embryos, swimming-up larvae, and/or early juveniles were sampled for analysis.

### Ploidy level analyses

Peripheral blood was collected from the caudal vessel into a heparinized syringe [49], kept at 4°C, and processed immediately with flow cytometry (Partec CCA I; Partec GmbH, Münster, Germany). The ploidy of each adult fish was measured as relative DNA content in blood cells, using 4´,6-diamidino-2-phenylindole (DAPI) first separately for erythrocytes and spermatozoa, and then pooled [48]. Erythrocytes and spermatozoa of a functionally diploid *A. ruthenus* gave relative DNA content of 2n as the diploid and 1n as the haploid standard. Peripheral bloods of thirty samples of juveniles from each fertilization trial were processed for flow cytometry [50].

For image cytometry, slides were conventionally stained with Giemsa and inspected microscopically (Olympus BHS microscope NCSPlanApo 60x dry objective coupled to a 3CCD Sony DXC-9100P color camera). At least 100 erythrocyte nuclei per specimen were recorded and analyzed by Olympus MicroImage v. 4.0 software. Erythrocyte nuclear area (NA, μm^2^) was assessed following the protocol in Flajšhans [51]. The NA of functionally diploid *A. ruthenus* provided the diploid standard.

For karyotyping, metaphase chromosomes were prepared from leucocytes of peripheral blood [52] from all parental individuals. Blood was collected in a heparinized syringe (Zentiva) from the caudal vein and the syringe left in an upright position at 4°C overnight. The sedimented leucocytes and erythrocytes were cultured separately in complete medium (T 199 - Sigma, FBS Superior - Baria, Antibiotic Antimycotic Solution - Sigma, Kanamycin monosulfate - Sigma, LPS - Sigma, PHA H15 - Biomedica, Mercaptoethanol - Sigma) for 6 days at 20°C. The cell suspension was prepared routinely by harvesting cells after colchicine treatment, hypotonization, and fixation. A drop of the cell suspension was placed on a microscope slide, dried, and stained with 5% Giemsa stain buffered to pH 7.0. The chromosomes from five purebred juveniles and five hybrid juveniles from each fertilization trail were prepared according to Völker and Kulmann [53].

Metaphase chromosome plates were examined microscopically (Olympus AX 70) and recorded with an Olympus DP30VW digital camera. Karyotypes were arranged using Ikaros MetaSystems (Metasystems, Germany) software.

### Molecular analyses of mitochondrial DNA

The genomic DNA was extracted from fin-clips of all parental individuals using the NucleoSpin®tissue kit (MACHEREY-NAGEL). Amplification followed a standard PCR protocol to amplify a 620 bp mtDNA fragment of the *control region*[54]. The PCR reaction was carried out under the following conditions: 95°C for 120 s, 5 cycles of 95°C for 60 s, 53 °C for 60 s, and 72°C for 60 s; 30 cycles of 95°C for 30 s, 53°C for 45 s, and 72°C for 60 s; and a final extension at 72°C for 12 min. The PCR products were purified using NucleoSpin® Extract II (MACHEREY-NAGEL) and sequenced in both directions by Macrogen (Seoul, Korea). Sequences were aligned using Geneious 5.4 software [55] and BLASTed against the NCBI nucleotide collection using Mega-BLAST. This NCBI database contains previously published sequences of the control region for most sturgeon species (http://www.ncbi.nlm.nih.gov/).

### Fragmentation analyses of microsatellites

The genomic DNA from twenty two swimming-up larvae fixed in 96% ethanol from each trial, as well as from fin-clips of all parent fish was extracted using the NucleoSpin®tissue kit (MACHEREY-NAGEL). Microsatellite DNA fingerprinting of seven microsatellite loci, *Afu19*, *Afu34*, *Afu39*, *Afu68*[56], *Spl101*, *Spl173*[57], and *Aox45*[58], was used. PCR were performed on a volume of 25 μl, containing 1 U Taq DNA polymerase, 10 pmol of each primer, 10–50 ng DNA, 100 μM of each dNTP, 2.5 mm MgCl_2_, and 2.5 μl 10 x incubation buffer. Amplifications were performed under the following conditions: one cycle at 94°C for 3min and 25 cycles at 94°C followed by locus-specific annealing conditions: 53°C for locus *Aox 45*; 57°C for loci *Afu 19 Afu 34 Afu 39, Afu 68, Spl 101* and *Spl 173*; followed by 72°C for 30s and a final extension at 72°C for 10min. The PCR products were inspected on agarose gel, then run in the ABI 3110 DNA analyzer. Genotypes were scored using GeneMapper v4.1 (Applied Biosystems, TM).

## Competing interests

The authors declare that they have no competing interests.

## Authors' contributions

MH carried out the genotyping and analyses of mtDNA, participated in fertilization experiments and ploidy level assessment by flow cytometry and wrote the manuscript. MHu conducted statistical analyses of mt DNA. PR provided and described karyological data and performed data quality check. MR contributed on karyological analyses and prepared Figure 4. DL participated on microsatellite analyses and calibrated microsatellite scoring. AL performed molecular data check and participated in microsatellite data scoring. MRo supervised artificial reproduction, participated in sperm characterization and evaluation of fertilization experiments. DG was responsible for breading of all parental fishes as well as all progeny. MP provided all sperm characteristics. DB carried out image cytometry slides and contributed to the flow cytometry analyses. MF designed the experiments, carried out flow cytomerty analyses, evaluated all data obtained from flow cytometry and image cytometry, participated in fertilization experimants and performed data check. All authors except MHu read and approved the final version of manuscript.

## Supplementary Material

Additional file 1 **Genotyping results observed at all analyzed loci for hybridization of *A. gueldenstaedtii *** **female and *A. baerii *** **spontaneous hexaploid male and of *A. baerii *** **female and *A. baerii *** **spontaneous hexaploid male.** Click here for file
