# Supplementary material for: Fertility of a spontaneous hexaploid male Siberian sturgeon, Acipenser baerii
Source: BMC Genet. 2014 Jan 10;15:5. doi: 10.1186/1471-2156-15-5 (PMC3893428; doi:10.1186/1471-2156-15-5)
Supplement: Additional file 1 — Genotyping results observed at all analyzed loci for hybridization of A. gueldenstaedtii female and A. baerii spontaneous hexaploid male and of A. baerii female and A. baerii spontaneous hexaploid male. [file 1471-2156-15-5-S1.pdf]

Sample	Marker	Allele 1	Allele 2	Allele 3	Allele 4
A. gueldenstaedtii female	Afu19		129	135	144
A. baerii hexaploid male	Afu19	120		135	
progeny 1	Afu19	120	129	135	144
progeny 2	Afu19	120		135	144
progeny 3	Afu19	120		135	144
progeny 4	Afu19	129		135	144
progeny 5	Afu19	120		135	144
progeny 6	Afu19	120		135	144
progeny 7	Afu19			135	144
progeny 8	Afu19			135	144
progeny 9	Afu19			135	144
progeny 10	Afu19	120		135	144
progeny 11	Afu19	120	129	135	144
progeny 12	Afu19	120	129	135	
progeny 13	Afu19	120		135	144
progeny 14	Afu19	120		135	144
progeny 15	Afu19	120		135	144
progeny 16	Afu19	120		135	144
progeny 17	Afu19	120	129	135	144
progeny 18	Afu19	120		135	144
progeny 19	Afu19	120	129	135	144
progeny 20	Afu19	120		135	144
progeny 21	Afu19	120	129	135	
progeny 22	Afu19	120	129	135	144

Sample	Marker	Allele 1	Allele 2	Allele3
A. gueldenstaedtii female	Afu34	130	136	139
A. baerii hexaploid male	Afu34	130	136	139
progeny 1	Afu34			
progeny 2	Afu34		136	139
progeny 3	Afu34	130	136	139
progeny 4	Afu34		136	139
progeny 5	Afu34	130	136	139
progeny 6	Afu34	130	136	139
progeny 7	Afu34	130	136	139
progeny 8	Afu34	130	136	139
progeny 9	Afu34	130	136	139
progeny 10	Afu34	130	136	139
progeny 11	Afu34	130	136	139
progeny 12	Afu34	130	136	139
progeny 13	Afu34	130	136	
progeny 14	Afu34	130	136	139
progeny 15	Afu34	130	136	139
progeny 16	Afu34		136	139
progeny 17	Afu34	130	136	139
progeny 18	Afu34		136	139
progeny 19	Afu34		136	139
progeny 20	Afu34		136	139
progeny 21	Afu34	130	136	139
progeny 22	Afu34		136	139

Sample	Marker	Allele 1	Allele2	Allele 3	Allele 4	Allele 5
A. gueldenstaedtii female	Afu39		122		128	150
A. baerii hexaploid male	Afu39	119		125	128	
progeny 1	Afu39		122		128	
progeny 2	Afu39			125	128	150
progeny 3	Afu39			125	128	150
progeny 4	Afu39	119		125	128	
progeny 5	Afu39			125	128	150
progeny 6	Afu39				128	
progeny 7	Afu39		122	125	128	
progeny 8	Afu39	119		125	128	150
progeny 9	Afu39		122	125	128	
progeny 10	Afu39		122	125	128	
progeny 11	Afu39	119		125	128	
progeny 12	Afu39			125	128	150
progeny 13	Afu39	119		125	128	
progeny 14	Afu39	119	122	125	128	
progeny 15	Afu39		122	125	128	150
progeny 16	Afu39		122	125	128	150
progeny 17	Afu39	119	122	125	128	
progeny 18	Afu39		122	125	128	
progeny 19	Afu39		122	125	128	
progeny 20	Afu39	119		125	128	150
progeny 21	Afu39		122	125	128	
progeny 22	Afu39	119	122	125	128	

Sample	Marker	Allele 1	Allele 2	Allele 3	Allele 4	Allele 5	Allele 6	Allele 7	Allele 8	Allele 9
A. gueldenstaedtii female	Afu68	136			156		192			240
A. baerii hexaploid male	Afu68		140	152		180		200	232	
progeny 1	Afu68	136	140	152		180				240
progeny 2	Afu68				156			200	232	240
progeny 3	Afu68	136	140	152			192		232	
progeny 4	Afu68	136		152				200	232	240
progeny 5	Afu68		140	152	156	180				240
progeny 6	Afu68	136	140	152			192		232	
progeny 7	Afu68	136	140					200	232	240
progeny 8	Afu68			152	156	180			232	240
progeny 9	Afu68	136				180			232	240
progeny 10	Afu68			152	156	180		200		240
progeny 11	Afu68	136	140				192		232	
progeny 12	Afu68			152	156		192	200	232	
progeny 13	Afu68		140		156		192		232	
progeny 14	Afu68	136		152			192		232	
progeny 15	Afu68	136	140				192	200	232	
progeny 16	Afu68				156		192	200	232	
progeny 17	Afu68	136		152					232	240
progeny 18	Afu68	136	140			180	192	200		
progeny 19	Afu68	136		152		180	192		232	
progeny 20	Afu68	136	140			180	192		232	
progeny 21	Afu68	136				180			232	240
progeny 22	Afu68		140	152	156		192	200		

Sample	Marker	Allele 1	Allele 2	Allele 3	Allele 4	Allele 5	Allele 6	Allele 7	Allele 8	Allele 9
A. gueldenstaedtii female	Aox45	121				142	145			157
A. baerii hexaploid male	Aox45		124	127	136	142		148	151	
progeny 1	Aox45	121	124				145	148	151	
progeny 2	Aox45	121	124		136	142			151	
progeny 3	Aox45				136	142		148		157
progeny 4	Aox45			127		142		148		157
progeny 5	Aox45		124			142			151	157
progeny 6	Aox45	121	124	127			145		151	
progeny 7	Aox45	121	124				145	148	151	
progeny 8	Aox45		124				145	148	151	157
progeny 9	Aox45			127		142		148		157
progeny 10	Aox45		124	127		142	145			157
progeny 11	Aox45	121	124	127	136					157
progeny 12	Aox45	121	124			142			151	157
progeny 13	Aox45		124	127		142			151	157
progeny 14	Aox45	121	124		136	142			151	
progeny 15	Aox45		124		136	142				157
progeny 16	Aox45	121	124		136	142		148		
progeny 17	Aox45	121		127		142	145		151	
progeny 18	Aox45			127		142	145	148		157
progeny 19	Aox45	121	124		136	142			151	
progeny 20	Aox45	121	124	127			145		151	
progeny 21	Aox45		124	127		142			151	157
progeny 22	Aox45			127		142		148		157

Sample	Marker	Allele 1	Allele 2	Allele 3	Allele 4
A. gueldenstaedtii female	Spl101	296	300		308
A. baerii hexaploid male	Spl101		300	304	308
progeny 1	Spl101	296	300	304	308
progeny 2	Spl101	296	300	304	308
progeny 3	Spl101	296	300	304	308
progeny 4	Spl101	296	300	304	308
progeny 5	Spl101	296	300	304	308
progeny 6	Spl101	296	300	304	308
progeny 7	Spl101	296	300	304	308
progeny 8	Spl101		300	304	308
progeny 9	Spl101	296	300	304	308
progeny 10	Spl101	296		304	308
progeny 11	Spl101	296	300	304	308
progeny 12	Spl101	296	300	304	
progeny 13	Spl101	296	300	304	308
progeny 14	Spl101	296	300	304	308
progeny 15	Spl101	296	300	304	308
progeny 16	Spl101	296	300	304	308
progeny 17	Spl101	296	300	304	308
progeny 18	Spl101	296	300	304	308
progeny 19	Spl101	296	300	304	308
progeny 20	Spl101	296	300	304	308
progeny 21	Spl101	296	300	304	308
progeny 22	Spl101	296	300	304	308

Sample	Marker	Allele 1	Allele 2	Allele 3	Allele 4	Allele 5	Allele 6	Allele 7
A. gueldenstaedtii female	Spl173		228	232	236			248
A. baerii hexaploid male	Spl173	224			236	240	244	
progeny 1	Spl173	224		232	236	240	244	
progeny 2	Spl173	224	228	232		240	244	
progeny 3	Spl173	224	228		236	240		
progeny 4	Spl173	224	228		236		244	248
progeny 5	Spl173		228	232	236	240	244	
progeny 6	Spl173			232	236	240	244	
progeny 7	Spl173			232	236	240		
progeny 8	Spl173	224			236	240	244	248
progeny 9	Spl173	224		232	236	240		
progeny 10	Spl173	224	228		236	240		248
progeny 11	Spl173	224		232		240	244	248
progeny 12	Spl173			232	236	240	244	
progeny 13	Spl173	224	228		236	240		
progeny 14	Spl173				236	240	244	248
progeny 15	Spl173	224		232	236	240		
progeny 16	Spl173	224	228		236	240	244	
progeny 17	Spl173	224	228		236	240	244	
progeny 18	Spl173	224	228	232	236	240		
progeny 19	Spl173		228		236	240	244	
progeny 20	Spl173		228		236	240	244	248
progeny 21	Spl173			232	236	240	244	
progeny 22	Spl173	224		232	236	240		248

Sample	Marker	Allele 1	Allele 2
A. baerii female	Afu19		135
A. baerii hexaploid male	Afu19	120	135
progeny 1	Afu19	120	135
progeny 2	Afu19	120	135
progeny 3	Afu19		135
progeny 4	Afu19	120	135
progeny 5	Afu19	120	135
progeny 6	Afu19	120	135
progeny 7	Afu19	120	135
progeny 8	Afu19	120	135
progeny 9	Afu19	120	135
progeny 10	Afu19		135
progeny 11	Afu19		135
progeny 12	Afu19		135
progeny 13	Afu19		135
progeny 14	Afu19		135
progeny 15	Afu19	120	135
progeny 16	Afu19	120	135
progeny 17	Afu19	120	135
progeny 18	Afu19	120	135
progeny 19	Afu19	120	135
progeny 20	Afu19	120	135
progeny 21	Afu19	120	135
progeny 22	Afu19	120	135

Sample	Marker	Allele 1	Allele 2	Allele 3
A. baerii female	Afu34	130	136	
A. baerii hexaploid male	Afu34	130	136	139
progeny 1	Afu34		136	139
progeny 2	Afu34	130	136	139
progeny 3	Afu34	130	136	139
progeny 4	Afu34		136	139
progeny 5	Afu34	130	136	139
progeny 6	Afu34		136	139
progeny 7	Afu34	130	136	139
progeny 8	Afu34	130	136	139
progeny 9	Afu34	130	136	139
progeny 10	Afu34		136	139
progeny 11	Afu34	130	136	139
progeny 12	Afu34	130	136	139
progeny 13	Afu34		136	139
progeny 14	Afu34	130	136	139
progeny 15	Afu34	130	136	139
progeny 16	Afu34	130	136	139
progeny 17	Afu34	130	136	139
progeny 18	Afu34		136	139
progeny 19	Afu34	130	136	139
progeny 20	Afu34	130	136	139
progeny 21	Afu34		136	139
progeny 22	Afu34	130	136	139

Sample	Marker	Allele 1	Allele 2	Allele 3	Allele 4
A. baerii female	Afu39		125	128	144
A. baerii hexaploid male	Afu39	119	125	128	
progeny 1	Afu39	119	125	128	
progeny 2	Afu39		125	128	144
progeny 3	Afu39		125	128	144
progeny 4	Afu39		125	128	144
progeny 5	Afu39		125	128	144
progeny 6	Afu39		125	128	144
progeny 7	Afu39		125	128	
progeny 8	Afu39		125	128	144
progeny 9	Afu39	119	125	128	
progeny 10	Afu39	119	125	128	144
progeny 11	Afu39		125	128	144
progeny 12	Afu39		125	128	
progeny 13	Afu39	119	125	128	
progeny 14	Afu39	119	125	128	144
progeny 15	Afu39		125	128	144
progeny 16	Afu39	119	125	128	
progeny 17	Afu39	119	125	128	144
progeny 18	Afu39		125	128	144
progeny 19	Afu39	119	125	128	144
progeny 20	Afu39	119	125	128	144
progeny 21	Afu39		125	128	
progeny 22	Afu39	119	125	128	144

Sample	Marker	Allele 1	Allele 2	Allele 3	Allele 4	Allele 5	Allele 6
A. baerii female	Afu68		152	160		200	232
A. baerii hexaploid male	Afu68	140	152		180	200	232
progeny 1	Afu68	140	152		180	200	
progeny 2	Afu68	140		160	180	200	
progeny 3	Afu68		152		180		232
progeny 4	Afu68		152	160		200	232
progeny 5	Afu68	140	152	160	180	200	232
progeny 6	Afu68		152	160			232
progeny 7	Afu68	140		160		200	232
progeny 8	Afu68		152			200	232
progeny 9	Afu68	140		160	180		232
progeny 10	Afu68			160	180		232
progeny 11	Afu68	140	152			200	232
progeny 12	Afu68		152	160	180	200	232
progeny 13	Afu68	140	152				232
progeny 14	Afu68	140	152	160		200	232
progeny 15	Afu68	140	152			200	232
progeny 16	Afu68	140	152	160			232
progeny 17	Afu68		152			200	232
progeny 18	Afu68	140	152				232
progeny 19	Afu68	140	152		180		232
progeny 20	Afu68	140	152	160		200	232
progeny 21	Afu68			160		200	232
progeny 22	Afu68	140	152	160	180	200	232

Sample	Marker	Allele 1	Allele 2	Allele 3	Allele 4	Allele 5	Allele 6	Allele 7
A. baerii female	Aox45		127	130			148	
A. baerii hexaploid male	Aox45	124	127		136	142	148	151
progeny 1	Aox45	124		130		142	148	151
progeny 2	Aox45		127		136	142	148	151
progeny 3	Aox45	124		130		142	148	151
progeny 4	Aox45	124	127	130	136	142		
progeny 5	Aox45		127	130		142	148	
progeny 6	Aox45		127		136	142	148	
progeny 7	Aox45	124	127	130	136		148	
progeny 8	Aox45	124	127				148	151
progeny 9	Aox45		127		136	142	148	
progeny 10	Aox45		127			142	148	
progeny 11	Aox45		127	130		142	148	
progeny 12	Aox45	124		130	136		148	
progeny 13	Aox45		127		136		148	
progeny 14	Aox45			130	136	142	148	
progeny 15	Aox45		127		136		148	
progeny 16	Aox45		127	130	136		148	
progeny 17	Aox45		127	130	136		148	
progeny 18	Aox45		127	130	136		148	
progeny 19	Aox45		127			142	148	151
progeny 20	Aox45	124	127	130	136		148	
progeny 21	Aox45	124		130			148	151
progeny 22	Aox45	124	127	130	136		148	

Sample	Marker	Allele 1	Allele 2	Allele 3	Allele 4	Allele 5
A. baerii female	Spl101	284			308	320
A. baerii hexaploid male	Spl101		300	304	308	
progeny 1	Spl101			304	308	320
progeny 2	Spl101	284	300		308	
progeny 3	Spl101			304	308	320
progeny 4	Spl101		300		308	320
progeny 5	Spl101	284	300	304	308	320
progeny 6	Spl101				308	320
progeny 7	Spl101	284	300	304	308	320
progeny 8	Spl101		300		308	320
progeny 9	Spl101	284	300	304	308	320
progeny 10	Spl101		300	304	308	320
progeny 11	Spl101		300	304	308	320
progeny 12	Spl101				308	320
progeny 13	Spl101			304	308	320
progeny 14	Spl101			304	308	320
progeny 15	Spl101				308	320
progeny 16	Spl101		300	304	308	320
progeny 17	Spl101	284	300		308	320
progeny 18	Spl101			304	308	320
progeny 19	Spl101	284	300	304	308	320
progeny 20	Spl101	284	300		308	320
progeny 21	Spl101		300		308	320
progeny 22	Spl101	284	300		308	320

Sample	Marker	Allele 1	Allele 2	Allele 3	Allele 4	Allele 5
A. baerii female	Spl173	224	236	240		252
A. baerii hexaploid male	Spl173	224	236	240	244	
progeny 1	Spl173	224	236	240		
progeny 2	Spl173	224	236	240	244	
progeny 3	Spl173		236	240	244	
progeny 4	Spl173	224		240	244	
progeny 5	Spl173	224	236	240	244	
progeny 6	Spl173	224	236	240		
progeny 7	Spl173	224	236	240		252
progeny 8	Spl173	224	236	240		
progeny 9	Spl173	224	236	240	244	
progeny 10	Spl173	224		240		252
progeny 11	Spl173		236	240	244	
progeny 12	Spl173	224	236	240	244	
progeny 13	Spl173		236	240	244	252
progeny 14	Spl173	224	236	240		252
progeny 15	Spl173	224	236	240	244	252
progeny 16	Spl173	224	236	240	244	
progeny 17	Spl173	224	236	240		
progeny 18	Spl173	224	236	240		252
progeny 19	Spl173	224		240		252
progeny 20	Spl173	224	236	240	244	252
progeny 21	Spl173	224	236	240	244	
progeny 22	Spl173	224	236	240	244	
